# Hope for happiness in an emerging country during the COVID-19 pandemic

**DOI:** 10.1186/s40359-026-05060-w

**Published:** 2026-06-23

**Authors:** Seher Nur Sulku, Levent Gul, Yagmur Kaya, Kubra Cosar

**Affiliations:** 1 Department of Econometrics, Ankara Haci Bayram Veli University, Ankara, 06500 Türkiye; 2 Department of Econometrics, Kirikkale University, Kirikkale, 71450 Türkiye

**Keywords:** COVID-19, Subjective well-being, Happiness, Multinomial logistic model

## Abstract

**Background:**

The coronavirus disease 2019 (COVID-19) pandemic created substantial social, economic, and psychological challenges worldwide. This study examines changes in subjective well-being and happiness patterns during the COVID-19 period compared with the pre-pandemic period in Türkiye as an emerging country context.

**Methods:**

We analyze nationally representative Life Satisfaction Survey data from the Turkish Statistical Institute (TurkStat) for the 2019–2021 period, covering approximately 10 thousand individuals aged 18 and above each year. Multinomial logistic regression models are employed for each year, preserving the original five-category happiness variable ranging from “very happy” to “very unhappy”.

**Results:**

First, during the COVID-19 period, the percentage of individuals who defined themselves as happy or very happy decreased from approximately 54% in 2019 to 48.7% in 2020, before slightly recovering to 50.1% in 2021. Second, “health” remained the most frequently reported source of happiness among categories such as success, job, love, and money both before and during the pandemic period. The findings indicate that the main determinants of happiness remained broadly stable during the pandemic-period years, although the magnitude of several associations changed. Women reported higher happiness levels than men, whereas employed women exhibited relatively lower happiness levels. Having a job alone did not consistently affect happiness; however, employed individuals with at least a high school education tended to report higher happiness levels before the pandemic period. Furthermore, higher income, being married, health satisfaction, income satisfaction, and social life satisfaction were positively associated with happiness. A U-shaped relationship between happiness and age groups was also observed. Although higher-income individuals consistently reported greater happiness levels, the strength of this association weakened during the pandemic-period years. Finally, hopefulness about the future demonstrated the strongest and most consistent association with happiness. Hopeful individuals exhibited substantially greater odds of belonging to the “very happy” category, with odds ratios of approximately 18.0 in 2019, 15.1 in 2020, and 17.9 in 2021.

**Conclusion:**

The findings suggest that although happiness levels declined during the COVID-19 period in Türkiye, the main determinants of happiness remained largely stable. Socioeconomic conditions, health satisfaction, and future expectations were important factors associated with happiness, while hopefulness about the future emerged as the strongest and most consistent predictor across all years.

**Supplementary Information:**

The online version contains supplementary material available at https://doi.org/10.1186/s40359-026-05060-w.

## Background

The coronavirus disease 2019 (COVID-19) pandemic profoundly disrupted daily life worldwide. Beyond its direct effects on physical health, the pandemic caused widespread social, economic, and psychological consequences through lockdowns, social distancing measures, economic uncertainty, and disruptions to work and social life [1, 2]. The COVID-19 outbreak has influenced the cognitive well-being of every individual [3, 4]. Beside fear, COVID-19 precautions such as social distancing rules and lockdowns, uncertainty, job insecurity, changes in people’s financial stability, negatively affected the mental health of individuals, and caused anxiety, depression and even suicides [4–9]. The well-being literature suggests that these pandemic-related disruptions may influence subjective well-being through multiple channels, including health concerns, social isolation, economic uncertainty, financial instability, and changes in future expectations [5, 7, 8]. Consistent with this perspective, Foa et al. [10] reported that pandemic severity and related restrictions were associated with changes in life satisfaction and emotional well-being during the COVID-19 period. Accordingly, understanding how happiness and subjective well-being evolved during the pandemic has become an important area of research.

Subjective well-being, often referred to as happiness, has been conceptualized from both hedonic and eudaimonic perspectives [11]. The hedonic perspective emphasizes pleasure attainment, positive emotions, and life satisfaction, whereas the eudaimonic perspective focuses on meaning, personal growth, optimism, and psychological functioning [12–19]. Beyond these conceptual perspectives, happiness is also shaped by psychological mechanisms such as social comparison processes, which suggest that individuals’ self-perceptions and self-evaluations are influenced by comparisons with others. In everyday life, social interactions and media exposure continuously provide information about other people’s achievements, behaviors, and lifestyles, which may affect individuals’ subjective well-being [20]. In addition, adaptation theory and the set-point theory of happiness suggest that individuals may psychologically adjust to favorable or unfavorable life conditions over time and tend to return to a relatively stable level of well-being despite external changes [21]. Positive emotions and psychological resilience may also play an important role in sustaining happiness over time, as resilient individuals may better cope with adverse conditions and maintain higher levels of subjective well-being and life satisfaction [22]. While psychological mechanisms determine an individual’s outlook on life and long-term happiness, their short-term levels of happiness, in particular, can be influenced by external factors. There are many factors affecting happiness that may differ between individuals and nations, geographical regions or communities with different value judgments [13, 14, 23–25]. Although happiness is directly related to an individual’s health, it is also affected by external factors such as the environment, place, time, income level, and overall well-being [13, 15, 24]. These factors determine people’s perspective on life and affect their level of happiness. Furthermore, studies on the determinants of happiness identify the socioeconomic status of a country as an important factor in addition to individuals’ socioeconomic factors [13–15, 24, 25]. Happiness is strongly related to the economic development of a country and increases with economic growth [26].

Recent studies have shown that both happiness levels and the factors associated with happiness changed during the pandemic period [4, 27–29]. For example, Bakkeli [30] identified the relationship between self-reported health and life satisfaction before and during the COVID-19 pandemic in Norway and examined the role of work in explaining health–life satisfaction. Delhey et al. [9] examined life satisfaction during the COVID-19 pandemic while considering the role of human, economic, social, and psychological capital via panel data from Germany and the United Kingdom for the [2020, 2021] period. Rossouw et al. [27] tracked the evolution of happiness before and during the COVID-19 pandemic via a Markov switching dynamic regression model for New Zealand. Mureşan et al. [4] attempted to identify differences in the cognitive well-being of individuals before, during and after the COVID-19 pandemic period in terms of future expectations via ANOVA and GLM methodologies for a sample of 1572 respondents from 43 countries worldwide from May 2020 to July 2021. Their study does not represent a specific country but concludes that an individual’s happiness level was influenced by the changes in personal and/or family income levels. Indeed, even if the decreased level of happiness may rise after the pandemic, it would not be alike before when people did not know about the virus.

Our study contributes to the literature examining the evaluation of happiness during the COVID-19 pandemic compared with that during the pre-pandemic period as well as the demographic factors that may interact as determinants for an emerging country, Türkiye. Even though Türkiye is an upper-middle income country, its rank is 112, just below Ghana; in 2022, world happiness was reported among 146 countries [31]. The literature on well-being research has reached a consensus that resource-rich individuals are happier with their lives than resource-poor individuals are [9]; thus, its contradictory circumstances make studying the Türkiye case even more interesting. We consider the nationally representative life satisfaction surveys (LSSs) of the Turkish Statistical Institute (TurkStat) for the pre-COVID-19 years of 2019 and the COVID-19 period of 2020–2021, with a sample size of approximately 10 thousand for each year. TurkStat has collected data on the cognitive happiness of 18 + aged noninstitutional populations since 2003 via the LSS; in the surveys, the subjective well-being as being happy is questioned when someone thinks about her/his life as a whole and feels how much happiness on a Likert scale. Thus, multinomial logit models are estimated separately for each year over the 2019–2021 period, since happiness, the outcome variable of interest, has multiple categories. The models additionally include a set of covariates selected based on the relevant literature and data availability. However, the LSS does not contain individual-level information on direct COVID-19 infection status or personal exposure to the virus. Therefore, our study does not aim to identify the causal effect of COVID-19 infection at the individual level. Rather, the years 2020 and 2021 are interpreted as representing the broader COVID-19 era in Türkiye, characterized by nationwide public health measures, economic uncertainty, and social disruptions. Accordingly, our findings should be interpreted as descriptive and associative comparisons between the pre-pandemic and pandemic-period years rather than as causal estimates of pandemic exposure.

For the Türkiye case, three studies have examined the concept of well-being during the COVID-19 pandemic [32–34]. These studies specifically focus on how the fear of the pandemic affects people’s well-being and how individuals may deal with it, and each collects data via their own web-based surveys shared on the internet, which are limited and very small in sample size compared with the LSS of TurkStat used in our study.

The remainder of the paper is organized as follows. First, the COVID-19 pandemic experience in Türkiye is discussed to provide contextual background for the analysis. Next, the literature on the determinants of happiness and subjective well-being is reviewed. The subsequent section introduces the data, variables, and multinomial logistic regression methodology employed in the study. The empirical findings are then presented and discussed, followed by a discussion of the study limitations. Finally, the paper concludes with the main findings and implications of the study.

### The Covid-19 pandemic experience in Türkiye

The first confirmed COVID-19 case in Türkiye was reported on March 11, 2020. Following this date, numerous preventive measures and public health policies were planned and implemented to combat the spread of COVID-19 as effectively as possible. Even before the first confirmed COVID-19 case was reported in Türkiye, the Coronavirus Scientific Advisory Board was established under the Ministry of Health on January 10, 2020. Following the identification of the first case, preventive measures, contact tracing practices, protection strategies, and treatment interventions were implemented nationwide in accordance with the national guidelines prepared by the Ministry of Health Pandemic Scientific Advisory Board [2].

The initial priority was to contain the spread of the virus. Accordingly, emergency measures were introduced, including the temporary suspension of in-person education for one week in primary and secondary schools and for three weeks in universities. Soon after, strict lockdown measures were implemented nationwide, including curfews, travel restrictions, and mandatory face mask policies in public spaces. Following the initial critical phase of the pandemic, the government announced a normalization roadmap on May 4, 2020. In line with this roadmap, a gradual normalization process was initiated through the phased relaxation of certain restrictions. Following this period, pandemic policies in Türkiye alternated between stricter containment measures and periods of relative reopening depending on the course of the outbreak. On January 13, 2021, a nationwide vaccination program was launched. As of June 1, 2022, Türkiye had fully transitioned into the normalization period following the COVID-19 pandemic restrictions [35].

In addition to its public health consequences, the pandemic also generated substantial economic and social disruptions in Türkiye. From an economic perspective, even before the COVID-19 pandemic, Türkiye experienced significant macroeconomic instability following the 2018 currency crisis, characterized by high inflation, exchange rate volatility, and increasing economic uncertainty [36, 37]. The pandemic emerged during a period in which the Turkish economy was already facing structural vulnerabilities, including rising unemployment, declining purchasing power, and widening socioeconomic inequalities. Following 2018, inflation in Türkiye increased dramatically, reaching nearly 85% in the final quarter of 2022 [38]. Meanwhile, the unemployment rate, which had remained around 10% prior to the pandemic, increased to approximately 13% during the COVID-19 period [39]. Moreover, restrictive public health measures also had substantial economic and social consequences in Türkiye. Economic activity slowed considerably in sectors such as tourism, transportation, retail trade, and hospitality, while labor market conditions became increasingly fragile due to disruptions in production processes and employment patterns. In addition, mobility restrictions and social distancing measures altered daily life and working conditions, accelerated the transition to remote working arrangements, and increased economic uncertainty for households and businesses. These economic and social disruptions may have amplified the psychological consequences of the pandemic and, consequently, affected individuals’ subjective well-being and happiness levels [40].

Türkiye’s experience with COVID-19 has followed a notable trajectory, both in terms of fluctuations in case and death counts and the scope of the vaccination campaign. The first confirmed COVID-19 case in Turkey was reported in March 2020, after which the country faced multiple waves of infection throughout 2020 and 2021. According to data from the Johns Hopkins Coronavirus Resource Center [41], as of October 2022, the cumulative number of confirmed cases in Türkiye had reached approximately 17 million, while COVID-19-related deaths had exceeded 100,000. Significant increases in case and death counts were observed particularly during the final months of 2020 and the first half of 2021, which placed additional strain on the healthcare system [41].

### Literature survey on determinants of happiness

Happiness and life satisfaction are essential aims of life for all people. At this point, how a better and happier life can be achieved is an attractive issue for many researchers. The concepts of happiness and life satisfaction are holistic matters that emerge under the influence and combination of many factors. The literature contains numerous studies regarding the direction and magnitude of the effects of these fundamental elements underlying happiness and life satisfaction.

An increase in the welfare level of individuals results in increased happiness. In this context, an increase in income level enhances people’s satisfaction levels in their lives [42–45]. However, while income influences individuals’ levels of happiness, it cannot be solely explained as a direct source of happiness [46–49]. Moreover, previous evidence from Türkiye indicates that relative income and individuals’ perceived position within society may influence happiness more strongly than absolute income itself. In this context, social comparison and expectations regarding future living standards also play a significant role in shaping subjective well-being [50]. In this respect, the differences in the characteristics of working life in the context of gender also hold significance when determining happiness. Within this framework, while working women appear to be less happy than non-working women, a broader examination considering gender shows that women generally tend to be happier than men [51–54]. Recent evidence from Türkiye also suggests that working women report lower happiness levels than homemakers, even after controlling for socioeconomic characteristics. In particular, home-related responsibilities and gender-based social pressures appear to contribute more strongly to the happiness disadvantage of working women than workplace-related problems [55]. Previous studies frequently emphasize that the sources of happiness differ between men and women, with men placing greater importance on achievement, career, and financial factors, whereas women tend to value health, love, and interpersonal relationships more highly [56].

Additionally, in studies focusing on happiness levels on the basis of educational attainment, an increase in educational level is found to decrease happiness levels. However, when the same studies asked participants about their employment status according to their educational level, it was found that individuals with higher education, when employed, had increased happiness levels compared with other groups [15, 23, 24, 57]. The level of individual freedom is also important for happiness and life satisfaction. In this context, when individuals’ happiness levels are measured on the basis of their marital status, it is understood that married individuals are happier than those who are not married and that they indicate that marriage does not impose restrictions on their freedom [48, 58–61].

In studies investigating how health status affects individual happiness, participants are asked about their health condition, and the answers obtained are then compared with their reported levels of happiness. In this context, it was found that people place health at the center of happiness. In the same studies, the happiness of participants was examined after they were divided into age groups, and a U-shaped relationship between happiness and age groups was observed. In other words, the likelihood of being happy decreases for middle-aged people compared to younger people, while the rate of happiness increases for older people compared to younger people [42, 46, 58, 62–64].

Studies conducted during the pandemic period revealed a significant decrease in people’s happiness levels, but these levels increased after the pandemic. Taking this into account, the determinants of happiness may have evolved during the pandemic. Moreover, happiness has a variable structure and is affected by phenomena such as time, space and process. Dramatic changes in concepts directly affect both how happiness is perceived by individuals and its level. In this context, the general happiness level has been negatively impacted by the numerous restrictions imposed on individuals’ lives and the concerns they have for their own health [65, 66].

Research comparing pre-pandemic and pandemic periods in terms of happiness revealed that in the Czech Republic, while the proportion of happy individuals was 68.4% before the pandemic, this rate decreased to 47.9% during the pandemic [67]. In the People’s Republic of China, the proportion of happy individuals decreased from 57.8% before the pandemic to 27.4% during the pandemic, especially due to the restrictions imposed [68]. Additionally, the link between health and happiness has also become more apparent. Especially during the pandemic, there was a significant convergence between health and happiness levels, but this relationship decreased after the end of the pandemic. A rise in happiness levels was observed in the elderly population after the pandemic, given their survival during the pandemic period [66, 69, 70]. There have also been significant changes in individuals’ working conditions during the COVID-19 pandemic. In particular, owing to work models such as working from home and flexible working, individuals’ work and personal lives have merged. In addition, many people have lost their jobs or faced the risk of unemployment. All of these factors have had a negative impact on people’s happiness [29, 65, 67, 68, 71–74]. Similarly, Gonzalez-Bernal et al. [75] reported that prolonged home confinement, insufficient information and social isolation during the COVID-19 pandemic significantly reduced individuals’ life satisfaction levels, whereas employment and access to outdoor spaces positively contributed to well-being. Furthermore, Okulicz-Kozaryn and Valente [76] showed that happiness levels in large urban areas declined more sharply during the pandemic than in rural areas because of higher infection risks and psychological distress in densely populated environments. Furthermore, in studies examining the relationship between happiness and being hopeful about the future, individuals who are hopeful about the future have higher happiness levels than those who are not [26, 54, 77].

In Türkiye, studies related to happiness have been conducted by Kahyaoğlu [78], Saygılı et al. [79], Satici et al. [32], Çebi Karaaslan et al. [80], Özmen et al. [33] and Peker et al. [34]. However, our study differs from the above in that it examines the determinants of happiness during the pandemic period compared with a prepandemic year. Özmen et al. [33] considered the relationship between the fear of COVID-19, well-being, and life satisfaction perceptions of adults—a sample of 3111 individuals via an online Google Forms survey—in Türkiye and found via regression analysis that COVID-19 fear was responsible for 11.3% and 1.3% of the total variance in well-being and life satisfaction, respectively, and that well-being accounted for 19.4% of the total variance in life satisfaction. Satici et al. [32] and Peker et al. [34] focused on the mental health of individuals and identified ways to address stress and fear caused by COVID-19. Satici et al. [32] defined a structural equation model on survey data of 971 Turkish adults from all cities that were collected via a web-based questionnaire shared on the internet to determine the relationships among resilience, hope, and subjective happiness to identify the mediating role of fear of COVID-19. They conclude that “individuals who are resistant to stress and have a belief that they can find a way to cope can help prevent the fear of COVID-19 and thus enhance good mental health”. Peker et al. [34] investigated how to reduce the negative impact of COVID-19 on mental health and reported that psychological resilience and coping with stress mitigate the effect of COVID-19 on stress and increase happiness levels on the basis of their online Google Forms survey of 827 adults only from Erzurum Province, Türkiye.

Many social, political, economic, legal, military, religious and cultural preventive measures have been taken to slow the spread of the pandemic in Türkiye, including the imposition of curfews in major cities, the establishment of social distancing measures, national and international travel restrictions, the closure of nonessential businesses, the suspension of collective religious ceremonies and the postponement of call-up, referral and discharge procedures in military barracks [81, 82]. All these measures and restrictions have significant implications for individuals’ lives and, consequently, their levels of happiness. Therefore, separate examinations are needed during the COVID-19 pandemic. The aim of this study is to introduce a unique perspective to the literature by focusing on the pre-pandemic and pandemic periods. In this context, the factors identified in the literature as foundational to happiness are analyzed for their impact on happiness, and the changes in these variables before and during the pandemic are assessed.

## Methods

### Data and variables

In this study we applied Turkish Statistical Institute (TurkStat)’s 2019, 2020 and 2021 Life Satisfaction Survey (LSS) Data with official permission. The micro data for the 2019, 2020 and 2021 LSS Data were prepared by Turkstat in accordance with the Regulation named “Regulation of Procedure and Principles of Data Confidentiality and Confident Data Security in Official Statistics” which was issued by the Article 13 of Statistics Law of Türkiye No. 5429 and entered into force on 20 June, 2006, following that of its publication in the Official Journal No: 26204. TURKSTAT guarantees the confidentiality of the information obtained is guaranteed in Law No. 5429.^1^

The LSS serves as a systematic tool for evaluating individuals’ overall happiness, social values, and general satisfaction with key life domains and public services. The survey facilitates the immediate measurement of satisfaction as well as the observation of trends over time. Because this study aims to demonstrate the impact of the COVID-19 pandemic, this secondary objective of monitoring longitudinal changes is particularly emphasized. In the 2019 LSS, representing the pre-pandemic period, 9,212 individuals participated; however, after accounting for 28 missing observations, data from 9,184 respondents were utilized in the analysis. Conversely, the pandemic and post-pandemic periods are represented by the 2020 and 2021 surveys, which included 10,103 and 10,073 participants, respectively, with no missing observations recorded in either year.

The variables applied in the analysis are summarized in Table 1. The original questions and responses from the LSS regarding the variables used in the analyses are provided in Appendix Table A.1. The distribution and percentage breakdown of the variables in relation to the happiness variable, within the categories defined in Table 1 for 2019, 2020, and 2021 are summarized in Appendix Tables A.2, A.3, and A.4, respectively.

**Table 1:** Categories of Dependent and Independent Variable

Variable	Category
Dependent Variable	
Happiness	1: Not happy at all
	2: Unhappy
	3: Neither happy nor unhappy
	4: Happy
	5: Very happy
Independent Variables	
Demographic variables	
Gender: Female	1: Female
	0: Male
Marital Status: Married	1: Married
	0: Not Married
Age Groups	1: 18–29
	2: 30–41
	3: 42–53
	4: 54–65
	5: +65
Geographic	
Settlement: Urban	1: Urban
	0: Other
Socioeconomic variables	
Job Status: Employed	1: Employed
	0: Not Employed
Education Level ≥ High school	1: High school and above
	0: Below high school
Settlement: Urban	1: Urban
	0: Other
Income (2019)	1: Q1 (0 – ₺2.015)
	2: Q2 (₺2.016 – ₺2.892)
	3: Q3 (₺2.893 – ₺4.049)
	4: Q4 (₺4.050 – ₺5.932)
	5: Q5 (₺5.933+)
Income (2020)	1: Q1 (0 – ₺2.229)
	2: Q2 (₺2.230 – ₺3.257)
	3: Q3 (₺3.258– ₺4.560)
	4: Q4 (₺4.561– ₺6.680)
	5: Q5 (₺6.681 +)
Income (2021)	1: Q1 (0 – ₺2.667)
	2: Q2 (₺2.668– ₺3.828)
	3: Q3 (₺3.829– ₺5.359)
	4: Q4 (₺5.360– ₺7.851)
	5: Q5 (₺7.851+)
Satisfaction	
Source of Happiness	1: Health
	0: Others (job, love, money, or other qualities)
Health Satisfaction:	1: Satisfied
	0: Not Satisfied
Income Satisfaction	1: Satisfied
	0: Not Satisfied
Social Life Satisfaction	1: Satisfied
	0: Not Satisfied
Being Hopeful about the Future	1: Hopeful
	0: Not Hopeful

Table 1 outlines the dependent and independent variables used in the study. To prevent potential information loss, the dependent variable (Happiness) was kept in its original survey format. The LSS measures subjective well-being as happiness via the question: “When you think about your life as a whole, how happy are you?” Responses are recorded on a five-category Likert scale ranging from (1) “very happy”, (2) “happy”, (3) “moderate”, (4) “unhappy”, to (5) “very unhappy”. The original five-category structure of the happiness variable was retained in order to preserve the full variability of the subjective well-being measure. Since the dependent variable, happiness, is categorical in nature, multinomial logit models were employed.

**Fig. 1:**
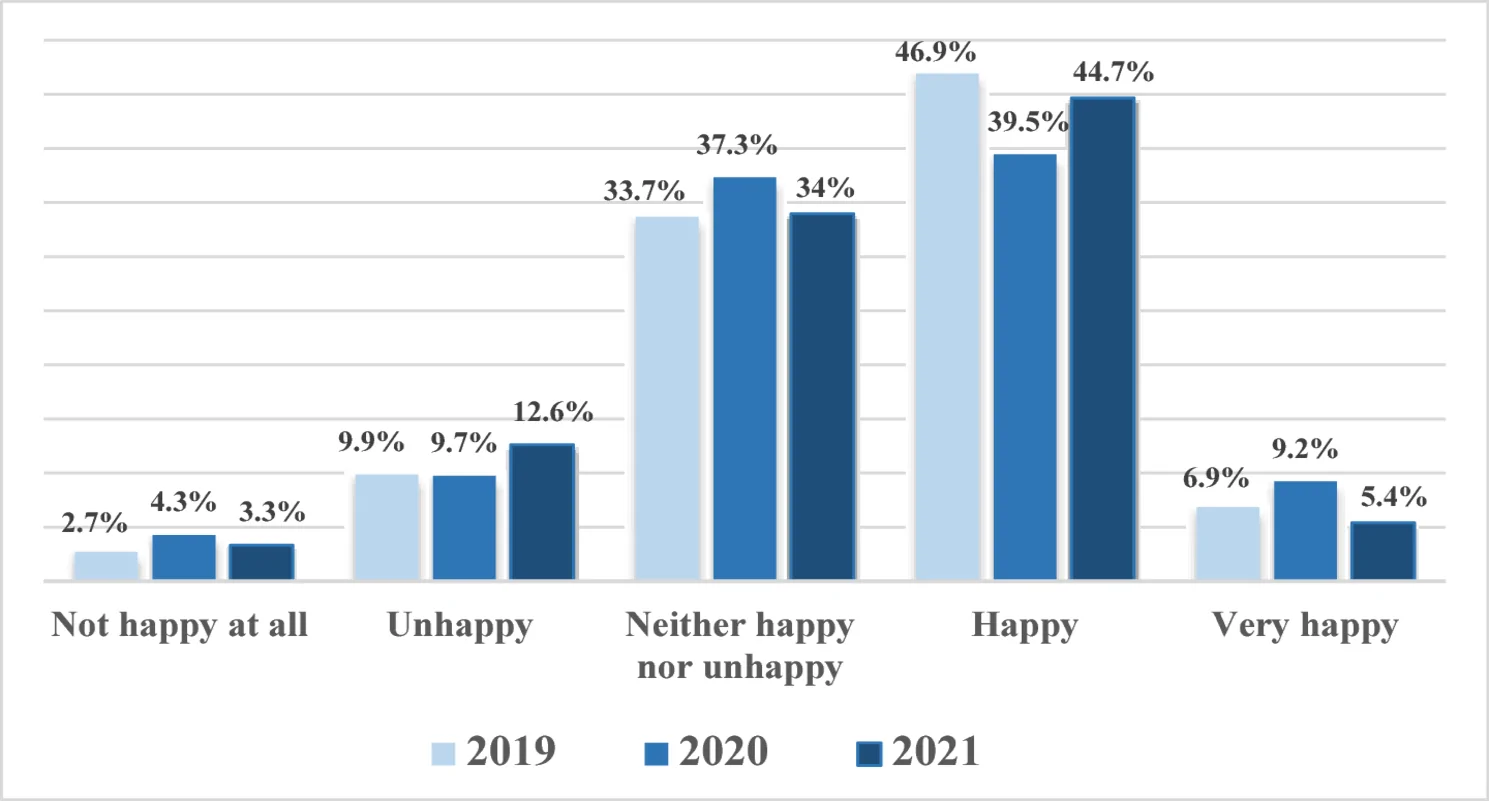
Distribution of Happiness. Sources: 2019, 2020 and 2021 Life Satisfaction Survey

The distribution of the dependent variable (Happiness) is presented in Fig. 1. Figure 1 illustrates the distribution of the happiness variable, revealing that responses are primarily clustered within the ‘undecided’ and ‘happy’ categories. While the largest percentage of participants reported being ‘happy,’ those selecting the extreme options constitute a minority. Notably, the proportion of respondents identifying as ‘unhappy’ increased in 2021. Conversely, the percentage of individuals reporting being ‘very happy’ decreased during the same year.

Many factors influence lifelong individual happiness, and they vary from person to person. Therefore, we have categorized twelve independent variables into four groups that allow for the observation of common effects and comparisons. These groups, consisting of categorical variables, comprise demographic, geographic, socioeconomic, and finally individual satisfaction variables.

Demographic variables: Gender takes a value of 1 if the individual is female and 0 if the individual is male; marital status takes a value of 1 if the individual is married and 0 otherwise; age takes a value of 1 if the individual’s age is between 18 and 29 years, 2 if between 30 and 41 years, 3 if between 42 and 53 years, 4 if between 54 and 65 years, and 5 if 65 years and over. The continuous age variable was categorized into specific age groups to better capture potential differences across young, middle-aged, and elderly populations during the COVID-19 period.

Geographic variable: Furthermore, the *Urban Settlement* variable was also included because densely populated urban areas were disproportionately affected by COVID-19 transmission and pandemic-related restrictions due to higher population mobility and social interaction, potentially influencing individuals’ subjective well-being during the pandemic period [1, 2, 42]. Since the LSS does not provide a direct urban–rural classification variable, an *Urban Settlement* dummy variable was constructed using the survey question: “Is your residence within the borders of a municipality/metropolitan municipality?”. The variable takes the value of 1 for respondents residing within municipality/metropolitan municipality borders and 0 otherwise. This approach is consistent with the broader literature that employs administrative settlement classifications and urbanisation proxies in analyses of well-being and spatial disparities.

Socioeconomic variables: Job status takes a value of 1 if the individual is employed and 0 otherwise; education level takes a value of 1 if the individual’s education level is high school and above and 0 otherwise (no school completed, primary school or secondary school). The income group variable takes different values for the years 2019, 2020, and 2021, as it varies annually. We construct five category income group variables from the 20% poorest (Q1) to the 20% richest (Q5). In 2019, household income takes 1 if the household income is between 0 and 2,015 TL (Turkish Lira), 2 if between 2,016 − 2,892 TL, 3 if between 2,893 and 4,049 TL, 4 if between 4,050 − 5,932 TL, and 5 if 5,933 TL and over. In 2020, household income takes a value of 1 if the household income is between 0 and 2,229 TL, 2 if between 2,230 and 3,257 TL, 3 if between 3,258 and 4,560 TL, 4 if between 4,561 and 6,680 TL, and 5 if 6,681 TL and over. In 2021, household income takes 1 if household income is between 0 and 2,667 TL, 2 if between 2,668 and 3,828 TL, 3 if between 3,829 and 5,359 TL, 4 if between 5,360 and 7,851 TL, and 5 if 7,852 TL and over.

Individual satisfaction variables: The source of happiness variable is constructed from the question “What makes you the happiest in your life?” and takes a value of 1 if the respondent identifies health as the main source of happiness, and 0 if the respondent identifies success, job, love, money, or other factors. The variables *Health Satisfaction*, *Income Satisfaction*, and *Social Life Satisfaction* are defined as binary indicators taking the value of 1 if the respondent reports being “very satisfied” or “satisfied” with the relevant domain, and 0 otherwise. The *Hopeful about the Future* variable is defined as a binary indicator taking the value of 1 if the respondent reports being “very hopeful” or “hopeful” about the future, and 0 otherwise. These variables also allow us to account for broader social and economic channels associated with the COVID-19 period, including social isolation, financial instability, and changes in future expectations that may affect subjective well-being.

**Fig. 2:**
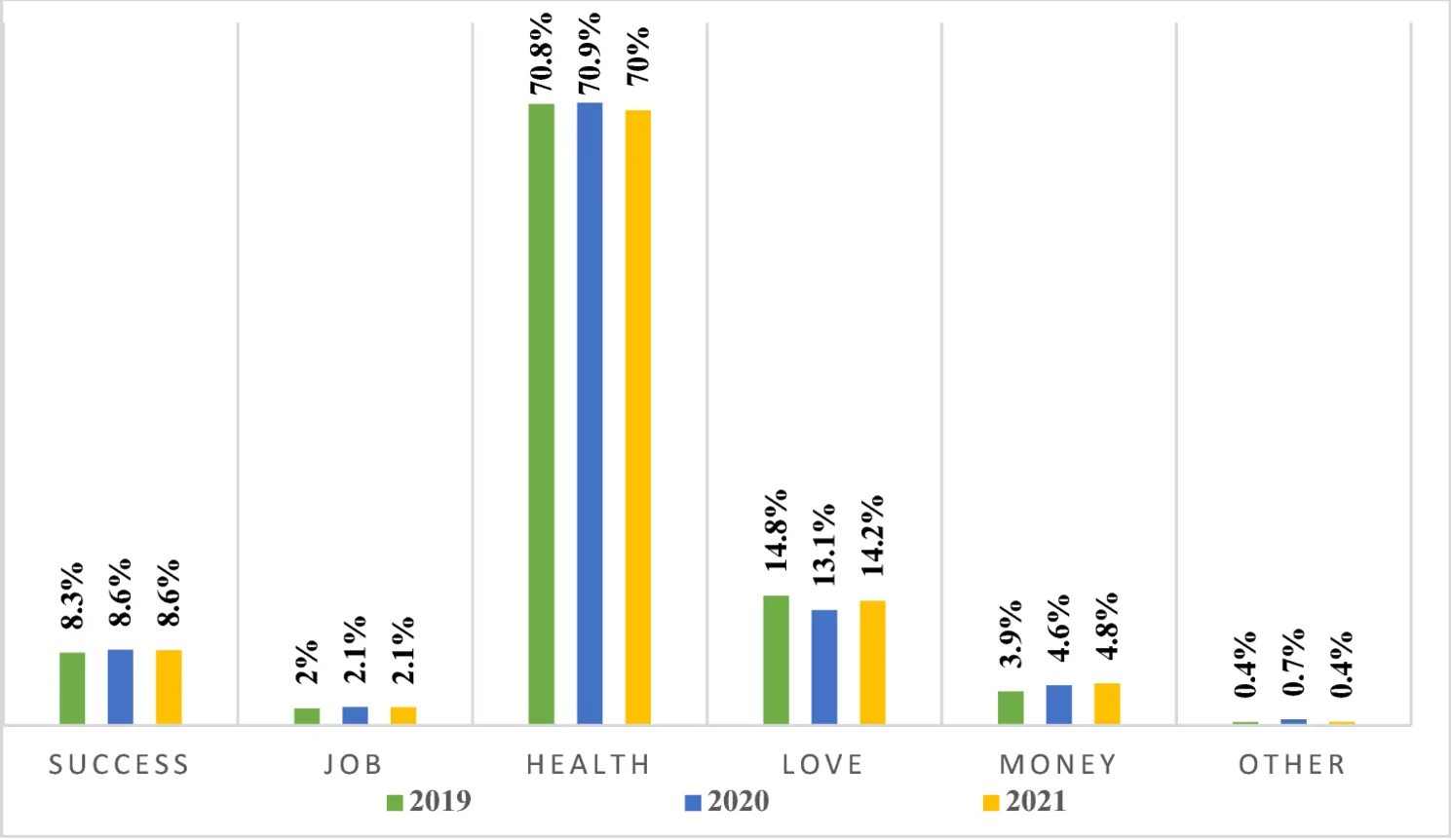
Distribution of the sources of happiness by year and category. Sources: 2019, 2020 and 2021 Life Satisfaction Survey

Finally, Fig. 2 presents the annual percentage distribution of the source of happiness variable.

The distribution of individuals’ sources of happiness for the [2019, 2021] period is presented in Fig. 2. Both before and during the COVID-19 pandemic, “health” was reported as the main source of happiness by the majority of Turkish respondents, with approximately 70% of individuals identifying health as their primary source of happiness, followed by “love” at approximately 14%.

### Statistical analysis

This study employs multinomial logistic regression models to examine subjective well-being and happiness patterns during the pre-pandemic year (2019) and the pandemic-period years (2020–2021) in Türkiye. Multinomial logistic regression is commonly used when the dependent variable consists of multiple categorical outcomes and greater flexibility is required in modeling heterogeneous response categories [83–85]. In this study, multinomial logit models were employed because the dependent variable, happiness, is measured as a five-category subjective well-being indicator ranging from “very happy” to “very unhappy.” Compared with ordered response models, the multinomial logit specification does not impose the proportional odds (parallel lines) assumption and allows category-specific comparisons across happiness levels. In the context of the multinomial logit model, the probability of an individual choosing alternative *j*$\:\in\:J$ is calculated as $\:P({Y}_{i}=j)=\frac{\mathrm{exp}\left({X}_{i}^{{\prime\:}}{\beta\:}_{j}\right)}{\sum\:_{k=1}^{J}\mathrm{exp}\left({X}_{i}^{{\prime\:}}{\beta\:}_{k}\right)}$, where the *X*_*i*_ vector of independent variables, $\:{\beta\:}_{j}$ coefficients specific to alternative *j*.

## Results

This study, which examines subjective well-being and happiness patterns during the pre-pandemic year (2019) and the pandemic-period years (2020–2021), estimated multinomial logistic regression models using Life Satisfaction Survey data. In the estimated models, the first category of the independent variables was established as the reference category (e.g., male for gender and unemployed for employment status). Similarly, the lowest happiness category, “Very unhappy”, was designated as the reference category for the dependent variable. A consistent reference-category structure was applied across all variables to facilitate interpretation of the multinomial logit estimates.

A total of 12 independent variables were included in the multinomial logistic regression models. Because the explanatory variables are categorical, a Polychoric correlation matrix was calculated to evaluate potential associations among variables and is presented in Appendix Tables A.5–A.7 for each year. In addition, Variance Inflation Factor (*VIF*) values are summarized in Appendix Table A.8. Evaluating the Polychoric correlation matrices together with the VIF statistics indicates that multicollinearity is not a concern in the estimated models. Accordingly, the multinomial logistic regression results are presented in Table 2^2^.

**Table 2:** Multinomial logistic outputs

Variable	2019		2020		2021	
	Odds ratio	*p*	Odds ratio	*p*	Odds ratio	*p*
Demographics						
Female	2.384	0.000^***^	3.197	0.000^***^	3.080	0.000^***^
Married	7.920	0.000^***^	4.615	0.000^***^	4.379	0.000^***^
Age Groups (*Reference: 18-29*)						
30-41	0.614	0.054^**^	0.832	0.339	1.268	0.293
42-53	0.432	0.001^***^	0.514	0.001^***^	0.860	0.536
54-65	0.434	0.004^***^	0.666	0.097^*^	0.742	0.272
+65	1.305	0.422	0.938	0.818	2.090	0.022^**^
Geographic						
Urban	0.792	0.534	1.162	0.547	0.952	0.887
Socioeconomic						
Employed	1.021	0.943	1.078	0.738	1.455	0.186
Education Level≥High school	0.764	0.276	1.072	0.716	1.070	0.765
Income (Lowest 20% income group)						
Q2	2.745	0.000^***^	1.478	0.028^**^	1.174	0.519
Q3	2.653	0.000^***^	1.561	0.036^**^	1.421	0.176
Q4	2.913	0.000^***^	1.918	0.003^***^	2.650	0.001^***^
Q5	7.707	0.000^***^	1.646	0.048^**^	2.765	0.000^***^
Satisfaction						
Health: Satisfied	4.406	0.000^***^	3.634	0.000^***^	3.699	0.000^***^
Income: Satisfied	3.262	0.000^***^	4.546	0.000^***^	4.371	0.000^***^
Social Life: Satisfied	7.939	0.000^***^	4.174	0.000^***^	5.077	0.000^***^
Interaction and Others						
Employed and ≥High school	1.878	0.074^*^	1.229	0.437	0.862	0.632
Employed and Female	0.848	0.645	0.513	0.022^**^	0.684	0.255
Source of Happiness: Health	2.004	0.000^***^	1.222	0.133	1.192	0.264
Hopeful about the Future: Hopeful	18.005	0.000^***^	15.126	0.000^***^	17.859	0.000^***^
Constant	0.004	0.000^***^	0.008	0.000^***^	0.004	0.000^***^
Demographics						
Female	2.578	0.000^***^	2.512	0.000^***^	3.280	0.000^***^
Married	4.611	0.000^***^	2.684	0.000^***^	2.735	0.000^***^
Age Groups (*Reference: 18-29*)						
30-41	0.778	0.266	1.002	0.990	1.405	0.067
42-53	0.649	0.059^*^	0.868	0.417	1.211	0.334
54-65	0.742	0.231	1.502	0.057^**^	1.394	0.127
+65	1.746	0.059^**^	2.259	0.001^***^	3.219	0.000^***^
Geographic						
Urban	0.684	0.217	0.724	0.124	0.690	0.160
Socioeconomic						
Employed	0.991	0.971	0.830	0.322	1.476	0.076**
Education Level≥High school	0.718	0.126	0.963	0.825	0.942	0.751
Income(Lowest 20% income group)						
Q2	1.900	0.002***	1.519	0.004***	0.930	0.680
Q3	1.419	0.072**	1.803	0.001***	1.007	0.970
Q4	1.743	0.018**	2.125	0.000***	1.676	0.019**
Q5	4.219	0.000***	1.675	0.021***	1.581	0.041**
Satisfaction						
Health: Satisfied	3.308	0.000***	3.434	0.000***	3.205	0.000***
Income: Satisfied	2.202	0.000***	2.814	0.000***	2.826	0.000***
Social Life: Satisfied	5.790	0.000***	3.166	0.000***	3.355	0.000***
Interaction and Others						
Employed and ≥High school	2.436	0.005***	1.727	0.019***	1.126	0.640
Employed and Female	0.667	0.196	0.727	0.202	0.588	0.051**
Source of Happiness: Health	2.253	0.000***	1.956	0.000***	1.700	0.000***
Hopeful about the Future: Hopeful	13.912	0.000***	10.042	0.000***	12.251	0.000***
Constant	0.143	0.000***	0.107	0.000***	0.167	0.000***
Demographics						
Female	1.537	0.020^**^	1.493	0.006^***^	1.860	0.000^***^
Married	2.646	0.000^***^	1.723	0.000^***^	1.590	0.001^***^
Age Groups (*Reference: 18-29*)						
30-41	0.903	0.647	0.975	0.877	1.255	0.208
42-53	0.866	0.521	0.849	0.326	1.161	0.439
54-65	0.962	0.876	1.413	0.094^**^	1.295	0.225
+65	1.554	0.130	1.261	0.327	2.130	0.004^***^
Geographic						
Urban	0.931	0.815	0.961	0.849	0.772	0.318
Socioeconomic						
Employed	0.985	0.950	0.888	0.509	1.546	0.040^**^
Education Level≥High school	0.839	0.411	1.210	0.244	1.106	0.581
Income (Lowest 20% income group)						
Q2	2.314	0.000^***^	1.547	0.002^***^	1.033	0.845
Q3	2.030	0.000^***^	1.981	0.000^***^	1.121	0.538
Q4	2.130	0.001^***^	2.346	0.000^***^	1.933	0.002^***^
Q5	5.046	0.000^***^	1.716	0.013^**^	1.735	0.012^**^
Satisfaction						
Health: Satisfied	1.808	0.000^***^	1.586	0.000^***^	1.558	0.001^***^
Income: Satisfied	1.220	0.364	1.413	0.042^**^	1.449	0.060^*^
Social Life: Satisfied	3.125	0.000^***^	1.756	0.000^***^	1.938	0.000^***^
Interaction and Others						
Employed and ≥High school	2.161	0.013**	1.200	0.416	0.902	0.679
Employed and Female	0.884	0.688	0.982	0.940	0.758	0.297
Source of Happiness: Health	1.794	0.000***	1.715	0.000***	1.506	0.001***
Hopeful about the Future: Hopeful	6.172	0.000***	4.849	0.000***	5.222	0.000***
Constant	0.570	0.150	0.753	0.284	1.126	0.708
Demographics						
Female	1.043	0.825	0.958	0.790	1.081	0.644
Married	1.921	0.000***	1.036	0.792	1.004	0.976
Age Groups (*Reference: 18-29*)						
30-41	1.095	0.698	1.295	0.153	1.284	0.191
42-53	0.984	0.949	1.326	0.129	1.491	0.048**
54-65	1.225	0.431	2.604	0.000***	1.379	0.150
+65	1.466	0.215	1.966	0.009***	1.631	0.078*
Geographic						
Urban	0.677	0.214	0.770	0.236	0.635	0.086*
Socioeconomic						
Employed	0.874	0.593	0.723	0.097**	1.134	0.567
Education Level≥High school	0.987	0.955	1.107	0.569	1.076	0.700
Income(Lowest 20% income group)						
Q2	1.586	0.030**	1.265	0.124	0.856	0.379
Q3	1.195	0.377	1.389	0.085*	0.785	0.215
Q4	1.179	0.504	1.553	0.032**	1.143	0.552
Q5	2.766	0.002***	1.409	0.149	1.012	0.958
Satisfaction						
Health: Satisfied	1.266	0.146	1.435	0.004***	1.218	0.144
Income: Satisfied	0.882	0.591	0.903	0.593	1.064	0.768
Social Life: Satisfied	2.772	0.000***	1.298	0.117	1.128	0.494
Interaction and Others						
Employed and ≥High school	1.589	0.155	1.484	0.111	0.836	0.494
Employed and Female	1.166	0.633	0.962	0.886	1.182	0.544
Source of Happiness: Health	1.464	0.013**	1.310	0.027**	1.234	0.109
Hopeful about the Future: Hopeful	1.868	0.001***	1.239	0.119	1.681	0.002***
Constant	1.233	0.603	1.120	0.688	3.059	0.001***
	2019		2020		2021	
Number of Observations	9184		10103		10073	
Log likelihood	-9817.249		-11431.966		-10877.514	
LR Chi-square	2981.960^***^		3726.39^***^		3607.77^***^	
Chi-square (Prob)	0.000		0.000		0.000	
Pseudo R2	0.131		0.140		0.142	

*. **. and *** denote statistical significance at the 1%. 5%. and 10% levels. respectively. In the multinomial logistic model, "Not happy at all" is used as the reference group. The 20% poorest (Q1) to the 20% richest (Q5)

The interpretation of multinomial logistic regression results requires evaluating how explanatory variables are associated with different happiness categories relative to the reference group (“Very unhappy”). In addition, comparisons across 2019, 2020, and 2021 provide descriptive evidence regarding changes in subjective well-being patterns during the pandemic-period years. However, these year-to-year differences should be interpreted as associative and descriptive comparisons rather than causal effects attributable solely to the COVID-19 pandemic.

Table 2 indicates that women reported higher levels of happiness relative to men across all years. In particular, women exhibited significantly greater odds of belonging to the “Very happy” and “Happy” categories relative to the “Very unhappy” reference group. The magnitude of these associations remained relatively stable during the pandemic-period years. For example, the odds ratio for women being classified as “Very happy” increased from 2.384 in 2019 to 3.197 in 2020 and remained high at 3.080 in 2021.

Married individuals also exhibited higher happiness levels relative to unmarried respondents. However, the strength of this association weakened during the pandemic-period years. For instance, the odds ratio for married individuals being classified as “Very happy” declined from 7.920 in 2019 to 4.615 in 2020 and 4.379 in 2021, although the association remained statistically significant across all years.

The age-group results suggest heterogeneous happiness patterns across demographic cohorts. Middle-aged individuals generally exhibited lower odds of belonging to the higher happiness categories relative to younger respondents aged 18–29, while individuals aged 65 and above tended to report higher happiness levels, particularly in the “Happy” category during 2020 and 2021. These findings are broadly consistent with the U-shaped happiness pattern frequently discussed in the well-being literature. However, the magnitude and statistical significance of the coefficients varied across happiness categories and years.

The Urban Settlement variable did not exhibit statistically significant effects in most model specifications. Nevertheless, the variable was retained because urban living conditions may capture differences associated with population density, social interaction patterns, and pandemic-related restrictions during the COVID-19 period.

Regarding socioeconomic variables, employment status did not exhibit a statistically significant relationship with happiness in most specifications prior to the pandemic period. However, in 2021 employed individuals displayed higher odds of belonging to the “Happy” and “Neither happy nor unhappy” categories. Educational attainment generally did not produce statistically significant effects after controlling for other demographic and socioeconomic variables.

Income displayed a strong positive association with happiness. Individuals belonging to higher income quintiles consistently reported greater odds of being in happier categories relative to respondents in the lowest income quintile. However, the magnitude of this association became weaker during the pandemic-period years. For example, in 2019 respondents in the highest income quintile (Q5) were 7.707 times more likely to report being “Very happy” relative to respondents in the lowest quintile (Q1), whereas this ratio declined to 1.646 in 2020 before partially recovering to 2.765 in 2021.

The satisfaction-related variables exhibited some of the strongest associations with happiness outcomes. Individuals satisfied with their health, income, and social life consistently demonstrated higher odds of belonging to the happier categories. Among these variables, satisfaction with social life displayed particularly strong associations before the pandemic period. For instance, respondents satisfied with their social life were approximately eight times more likely to report being “Very happy” in 2019. Although the magnitude of this association declined during 2020 and 2021, it remained statistically significant throughout the study period.

Similarly, respondents satisfied with their income and health also reported substantially higher happiness levels. Income satisfaction exhibited a relatively stronger association during the pandemic-period years, whereas the magnitude of the health satisfaction coefficient slightly declined compared with the pre-pandemic period.

The variable measuring hopefulness about the future demonstrated the strongest and most consistent association with happiness across all years and categories. Individuals who reported being hopeful about the future exhibited dramatically higher odds of belonging to the happier categories relative to individuals who were not hopeful. For example, hopeful respondents were approximately 18 times more likely to report being “Very happy” in 2019, and this strong positive association persisted during 2020 and 2021.

Respondents identifying health as their primary source of happiness also exhibited greater odds of belonging to happier categories relative to respondents identifying other sources of happiness. However, the magnitude of this association became weaker during the pandemic-period years, particularly for the “Very happy” category.

Finally, the interaction terms reveal heterogeneous patterns across demographic and socioeconomic groups. In particular, employed women exhibited relatively lower happiness odds during the pandemic-period years compared with non-employed women. Similarly, the positive association between employment and higher education weakened during the pandemic-period years and became statistically insignificant in 2021.

## Discussion

In this study, we investigate the factors associated with subjective well-being and happiness among individuals living in Türkiye and examine how happiness patterns changed during the pre-pandemic year (2019) and the pandemic-period years (2020–2021). Using nationally representative Life Satisfaction Survey (LSS) data, multinomial logistic regression models were estimated to evaluate the associations between demographic, socioeconomic, and individual satisfaction variables and different happiness categories. Accordingly, the findings should be interpreted as descriptive and associative comparisons across years rather than as causal effects attributable solely to the COVID-19 pandemic.

When examining the relationship between income and happiness, the findings indicate that higher-income individuals consistently exhibit higher levels of happiness compared with lower-income groups. This finding is consistent with the literature suggesting a positive relationship between income and subjective well-being (Linley and Burns [46], Lucas and Lawless [42], Rutledge et al. [43], and Jebb et al. [49]). However, the results also suggest that the magnitude of the association between income and happiness weakened during the pandemic-period years relative to the pre-pandemic period. In particular, the relative odds of belonging to the “Very happy” category among individuals in the highest income quintile declined substantially in 2020 and only partially recovered in 2021. This finding may reflect broader economic uncertainty, inflationary pressures, and social disruptions experienced during the pandemic period, which may have reduced the relative contribution of income to subjective well-being.

Regarding gender differences, women consistently reported higher happiness levels relative to men across all years. In particular, women exhibited significantly higher odds of belonging to the “Very happy” and “Happy” categories. These findings are consistent with studies reporting higher subjective well-being among women [51, 53]. However, the interaction results indicate that employed women exhibited relatively lower happiness levels compared with non-employed women, and this negative association became more visible during the pandemic-period years. These findings may reflect the additional social and economic burdens experienced by working women during the COVID-19 period, including work–family balance pressures and increased uncertainty in labor-market conditions.

Educational attainment did not exhibit a statistically significant independent relationship with happiness in most model specifications after controlling for demographic and socioeconomic factors. However, the interaction term between employment and higher education suggests that employed individuals with at least a high school education were more likely to belong to happier categories prior to the pandemic period, although this positive association weakened and became statistically insignificant by 2021. Therefore, the findings suggest that the relationship between education and happiness may operate indirectly through employment and socioeconomic conditions rather than through educational attainment alone. These findings partially differ from studies suggesting a direct relationship between education and happiness (Chow [57], Layard [23], and Diener and Biswas [15]) and may reflect differences in labor-market dynamics and socioeconomic uncertainty during the pandemic period.

Health-related variables demonstrated an important relationship with happiness outcomes. Individuals satisfied with their health consistently reported significantly greater odds of belonging to happier categories, supporting previous findings emphasizing the close relationship between perceived health and subjective well-being (Kahneman and Deaton [62], Linley and Burns [46], and Louck and Levy [66]). However, although health satisfaction remained positively associated with happiness throughout the study period, the magnitude of this association slightly weakened during the pandemic-period years. Similarly, respondents identifying “health” as their primary source of happiness also displayed higher happiness levels, although this association became less pronounced during 2020 and 2021. These findings may indicate that during periods of widespread uncertainty and social disruption, additional dimensions of well-being beyond health become increasingly important determinants of happiness.

Consistent with the well-being literature reporting a U-shaped relationship between age and happiness [29, 42, 58, 64, 66], middle-aged individuals generally exhibited lower happiness levels relative to younger respondents, whereas older individuals, particularly those aged 65 and above, tended to report higher happiness levels. The findings suggest that these patterns persisted during the pandemic-period years, although the magnitude and statistical significance of coefficients varied across happiness categories and years.

Among all explanatory variables, hopefulness about the future demonstrated the strongest and most consistent association with happiness outcomes. Individuals who reported being hopeful about the future exhibited dramatically higher odds of belonging to happier categories throughout all years of analysis. This finding is consistent with studies emphasizing hope as a protective factor for psychological well-being and subjective happiness during periods of uncertainty and crisis [32, 54, 86]. Although the magnitude of the estimated coefficients varied somewhat across years and happiness categories, hopefulness remained one of the strongest correlates of subjective well-being during both the pre-pandemic and pandemic-period years.

Finally, satisfaction with social life and income also exhibited strong positive associations with happiness. In particular, social life satisfaction demonstrated one of the strongest relationships with the “Very happy” category prior to the pandemic period, although the magnitude of this association declined during 2020 and 2021. This pattern may reflect the effects of social distancing measures, mobility restrictions, and reduced social interaction during the COVID-19 period. Similarly, income satisfaction remained strongly associated with happiness across all years and became relatively more important during the pandemic-period years.

## Limitations

This study has two main limitations. The first limitation is that the Life Satisfaction Survey (LSS) published by TurkStat does not include questions related to the COVID-19 process or potential pandemics. Therefore, personal satisfaction questions are utilized to reflect the pandemic period. These questions are as follows: “*What make you the happiest in your life?*” to indicate the source of happiness, “*Are you satisfied with your health?*” to quantify perceived health satisfaction, and “*How hopeful are you from your future?*” to reflect an individual’s future expectations. Furthermore, the LSS does not contain individual-level information regarding direct COVID-19 infection status or personal exposure to the virus. Therefore, our findings should be interpreted as descriptive and associative comparisons between the pre-pandemic and pandemic-period years rather than as causal estimates of pandemic exposure.

The second limitation is that the start date of the COVID-19 process in Türkiye was March 11, 2020. However, the COVID-19 process is still ongoing, which hinders evaluations from being classified as prepandemic or postpandemic. Hence, the years 2020 and 2021, during which the effects of the pandemic were most intensely felt, are considered pandemic periods. Model results are evaluated accordingly.

## Conclusion

This study examines happiness and its determinants in Türkiye before and during the COVID-19 pandemic using nationally representative Life Satisfaction Survey data for 2019–2021. During the pandemic period, the proportion of individuals reporting happiness declined from almost 54% in 2019 to approximately 50% on average. Health remained the most frequently reported source of happiness throughout the study period.

The main determinants of happiness remained broadly stable across the pre-pandemic and pandemic-period years, although the magnitude of several associations changed during the COVID-19 period. The findings also highlight the importance of eudaimonic well-being alongside hedonic dimensions of happiness. An individual’s level of hopefulness most significantly affects her/his level of happiness, with hopeful individuals exhibiting substantially greater odds of belonging to happier categories throughout the 2019–2021 period.

Individuals satisfied with their health consistently reported higher happiness levels throughout the study period, although the strength of this association weakened slightly during the pandemic years. Furthermore, the results support a U-shaped relationship between age and subjective well-being in Türkiye, with middle-aged individuals reporting lower happiness levels than younger and older age groups. These age-related patterns remained broadly stable during the pandemic-period years.

Higher-income individuals consistently exhibited greater odds of belonging to happier categories, although this relationship weakened during the pandemic period. In addition, although employment alone did not have a consistently significant effect on happiness, the interaction findings suggest that employed individuals with at least a high school education tended to report higher happiness levels, particularly before the pandemic; however, this association weakened by 2021. Married individuals also generally reported higher happiness levels.

Women appeared to be happier than men both before and during the pandemic. However, working women reported lower happiness levels than nonworking women and men, and this pattern became more pronounced during the pandemic period. This finding highlights the importance of further examining women’s well-being in both workplace and household settings.

Finally, satisfaction with social life and income exhibited strong positive associations with happiness throughout the study period. However, the association between social life satisfaction and happiness weakened during the pandemic years, highlighting the importance of social connectedness for subjective well-being.

This study contributes to the literature by providing evidence on subjective well-being in Türkiye during a major public health crisis. Accordingly, the findings should be interpreted as descriptive and associative comparisons across years rather than as causal estimates attributable solely to the COVID-19 pandemic.

## Supplementary Information

Supplementary Material 1: Table A. 1. Survey Instruments and Categorical Responses for Life Satisfaction Variables. Table A. 2. Distribution of Life Satisfaction by Happiness Categories in 2019. Table A. 3. Distribution of Life Satisfaction by Happiness Categories in 2020. Table A. 4. Distribution of Life Satisfaction by Happiness Categories in 2021. Table A. 5. Polychoric Correlation Matrix of Explanatory Variables (2019). Table A. 6. Polychoric Correlation Matrix of Explanatory Variables (2020). Table A. 7. Polychoric Correlation Matrix of Explanatory Variables (2021). Table A. 8. Variance Inflation Factors (VIF) for Explanatory Variables.

## Data Availability

In this study The Life Satisfaction Survey (LSS) of 2019, 2020 and 2021 have been used. The Life Satisfaction Survey is administered annually by the Turkish Statistical Institute (TurkStat). TurkStat prohibits the sharing of the data with third parties; thus, the authors cannot share it with third parties. The TurkStat 2019, 2020 and 2021 Life Satisfaction Survey Data cannot be copied or released to any other person or organization, as stated in Article 14 of the Statistics Law of Türkiye No. 5429. However, all the datasets are available from the corresponding author upon reasonable request with permission from third parties.

## Footnotes

1 TurkStat has its ethics committee and TurkStat’s Regulation on the Principles of Ethical Behavior of the Public Officials and Application Procedures and Essentials can be reached by https://www.tuik.gov.tr/Kurumsal/PDF_Detay. Respondents (human participants) in TurkStat surveys are selected from the Address Based Population Registration System using sampling techniques to represent all segments of the society by TurkStat. Every chosen “Respondents” have to participate in the TurkStat’ survey as Pursuant to Article 54 of the Turkish Statistics Law No. 5429, administrative fines are imposed in case the requested information is not provided in a timely, complete and accurate manner. The imposition of an administrative fine does not remove the respondent’s obligation to provide information.

2 Only odds ratio and *p values* are given in Table 2, but the coefficients, standard errors, and confidence intervals can be provided by the authors upon request.

## Abbreviations

ANOVA: Analysis of Variance
COVID-19: Coronavirus Disease 2019
GLM: Generalized Linear Model
LR: Likelihood Ratio
LSS: Life Satisfaction Survey
TL: Turkish Lira
TurkStat: Turkish Statistical Institute
VIF: Variance Inflation Factor
