# Supplementary material for: Hope for happiness in an emerging country during the COVID-19 pandemic
Source: BMC Psychol. 2026 Jun 23;14:1290. doi: 10.1186/s40359-026-05060-w (PMC13548595; doi:10.1186/s40359-026-05060-w)
Supplement: Supplementary file 1 [file 40359_2026_5060_MOESM1_ESM.docx]

**Appendix**

**Table A. 1.** Survey Instruments and Categorical Responses for Life Satisfaction Variables

| **Variable** | **Question** | **Category(Ordinal)** |
| --- | --- | --- |
| Happy | When you think your life as a whole. how happy are you? | Very happy |
|  |  | Happy |
|  |  | Neither happy nor unhappy |
|  |  | Unhappy |
|  |  | Not happy at all |
| Gender | Sex of the individual | Male |
|  |  | Female |
| Marital Status | What is your marital status? | Never married |
|  |  | Married |
|  |  | Divorced |
|  |  | Widowed |
| Job Status | In the last week. did you do any work for pay or profit in a job? | Worked |
|  |  | Did not worked but interest continues with work |
|  |  | Did not worked |
| Education Level | What is your completed level of education? (Will be answered on the basis of the highest diploma received) (Only one option can be marked) | No school completed |
|  |  | Primary school |
|  |  | General lower secondary school |
|  |  | Vocational and technical junior secondary school |
|  |  | Primary education |
|  |  | General high school |
|  |  | Vocational and technical high school |
|  |  | Post secondary of 2 or 3 years |
|  |  | Faculty of 4 years |
|  |  | Faculty of 5 or 6 years |
|  |  | Doctorate |
| Age | Individual's completed age | + 18 |
| Source of Happiness | What make you the most happy in your life? | Success |
|  |  | Job |
|  |  | Health |
|  |  | Love |
|  |  | Money |
|  |  | Other |
| Health Satisfaction | Are you satisfied with your health? | Very satisfied |
|  |  | Satisfied |
|  |  | Neither satisfied. nor unsatisfied |
|  |  | Not satisfied |
|  |  | Not satisfied at all |
| Income Satisfaction | Are you satisfied with your household monthly income? | Very satisfied |
|  |  | Satisfied |
|  |  | Neither satisfied. nor unsatisfied |
|  |  | Not satisfied |
|  |  | Not satisfied at all |
| Social Life Satisfaction | Are you satisfied with social life (entertainment. cultural and sporting activities. etc.)? | Very satisfied |
|  |  | Satisfied |
|  |  | Neither satisfied. nor unsatisfied |
|  |  | Not satisfied |
|  |  | Not satisfied at all |
| Hopeful about the Future | How hopeful are you from your future? | Very hopeful |
|  |  | Hopeful |
|  |  | Not hopeful |
|  |  | Not hopeful at all |
| Urban: Settlement | Is your residence in the borders of the municipality/metropolitan municipality? | Yes |
|  |  | No |
| Income^[1]^ | Which of the below interval represents the net utilized (salaries. wages. rent. unearned) income of your household? | 0–2 015 TL |
|  |  | 2 016 TL − 2 892 TL |
|  |  | 2 893 TL − 4 049 TL |
|  |  | 4 050 − 5 932 TL |
|  |  | 5 933 + TL |

**Table A. 2.** Distribution of Life Satisfaction by Happiness Categories in 2019

| **Variable Category** | **Not Happy at all** | **Unhappy** | **Neither Happy nor Unhappy** | **Happy** | **Very Happy** |
| --- | --- | --- | --- | --- | --- |
| **Gender** |  |  |  |  |  |
| Male | 126 (50.4%) | 468 (51.83%) | 1523 (49.27%) | 1815 (42.1%) | 275 (43.72%) |
| Female | 124 (49.6%) | 435 (48.17%) | 1568 (50.73%) | 2496 (57.9%) | 354 (56.28%) |
| **Marital Status** |  |  |  |  |  |
| Not married | 117 (46.8%) | 305 (33.78%) | 933 (30.18%) | 1032 (23.94%) | 120 (19.08%) |
| Married | 133 (53.2%) | 598 (66.22%) | 2158 (69.82%) | 3279 (76.06%) | 509 (80.92%) |
| **Age Groups** |  |  |  |  |  |
| 18–29 | 57 (22.8%) | 169 (18.72%) | 660 (21.35%) | 936 (21.71%) | 165 (26.23%) |
| 30–41 | 57 (22.8%) | 225 (24.92%) | 765 (24.75%) | 1061 (24.61%) | 171 (27.19%) |
| 42–53 | 64 (25.6%) | 213 (23.59%) | 752 (24.33%) | 924 (21.43%) | 129 (20.51%) |
| 54–65 | 47 (18.8%) | 197 (21.82%) | 573 (18.54%) | 747 (17.33%) | 86 (13.67%) |
| + 65 | 25 (10%) | 99 (10.96%) | 341 (11.03%) | 643 (14.92%) | 78 (12.4%) |
| **Settlement** |  |  |  |  |  |
| Rural | 14 (5.6%) | 67 (7.39%) | 144 (4.64%) | 265 (6.13%) | 27 (4.28%) |
| Urban | 236 (94.4%) | 840 (92.61%) | 2959 (95.36%) | 4.056 (93.87%) | 604 (95.72%) |
| **Job status** |  |  |  |  |  |
| Not employed | 157 (62.8%) | 529 (58.58%) | 1675 (54.19%) | 2584 (59.94%) | 349 (55.48%) |
| Employed | 93 (37.2%) | 374 (41.42%) | 1416 (45.81%) | 1727 (40.06%) | 280 (44.52%) |
| **Education Level** | |  |  |  |  |
| Below high school | 170 (68%) | 575 (63.68%) | 1825 (59.04%) | 2677 (62.1%) | 359 (57.07%) |
| High school and above | 80 (32%) | 328 (36.32%) | 1266 (40.96%) | 1634 (37.9%) | 270 (42.93%) |
| **Income Groups** | |  |  |  |  |
| Q1 | 102 (40.8%) | 277 (30.68%) | 589 (19.06%) | 941 (21.83%) | 77 (12.24%) |
| Q2 | 43 (17.2%) | 191 (21.15%) | 628 (20.32%) | 841 (19.51%) | 112 (17.81%) |
| Q3 | 58 (23.2%) | 194 (21.48%) | 781 (25.27%) | 916 (21.25%) | 161 (25.6%) |
| Q4 | 33 (13.2%) | 118 (13.07%) | 508 (16.43%) | 721 (16.72%) | 118 (18.76%) |
| Q5 | 14 (5.6%) | 123 (13.62%) | 585 (18.93%) | 892 (20.69%) | 161 (25.6%) |
| **Health Satisfaction** | |  |  |  |  |
| Not Satisfied | 149 (59.4%) | 480 (52.92%) | 1240 (39.96%) | 1068 (24.72%) | 102 (16.16%) |
| Satisfied | 101 (40.4%) | 427 (47.08%) | 1863 (60.04%) | 3253 (75.28%) | 529 (83.64%) |
| **Income Satisfaction** | |  |  |  |  |
| Not Satisfied | 220 (88%) | 757 (83.46%) | 2189 (70.54%) | 2078 (48.09%) | 213 (33.76%) |
| Satisfied | 30 (12%) | 150 (16.54%) | 914 (29.46%) | 2243 (51.91%) | 418 (66.24%) |
| **Social Life Satisfaction** | |  |  |  |  |
| Not Satisfied | 223 (89.2%) | 670 (73.87%) | 1967 (63.39%) | 1750 (40.5%) | 183 (29%) |
| Satisfied | 27 (10.8%) | 237 (26.13%) | 1136 (36.61%) | 2571 (59.5%) | 448 (71%) |
| **Source of Happiness** | |  |  |  |  |
| Others | 111 (44.4%) | 316 (34.99%) | 969 (31.35%) | 1107 (25.68%) | 182 (28.93%) |
| Health | 139 (55.6%) | 587 (65.01%) | 2122 (68.65%) | 3204 (74.32%) | 447 (71.07%) |
| **Hopeful about the Future** | |  |  |  |  |
| Not hopeful | 203 (81.2%) | 611 (67.66%) | 1131 (36.59%) | 701 (16.26%) | 72 (11.45%) |
| Hopeful | 47 (18.8%) | 292 (32.34%) | 1940 (63.41%) | 3610 (83.74%) | 557 (88.55%) |

**Table A. 3.** Distribution of Life Satisfaction by Happiness Categories in 2020

| **Variable Category** | **Not Happy at all** | **Unhappy** | **Neither Happy nor Unhappy** | **Happy** | **Very Happy** |
| --- | --- | --- | --- | --- | --- |
| **Gender** |  |  |  |  |  |
| Male | 257 (59.77%) | 591 (60.06%) | 1959 (51.96%) | 1791 (44.93%) | 405 (43.41%) |
| Female | 173 (40.23%) | 393 (39.94%) | 1811 (48.04%) | 2195 (55.07%) | 528 (56.59%) |
| **Marital Status** |  |  |  |  |  |
| Not married | 187 (43.49%) | 386 (39.23%) | 1224 (32.47%) | 1042 (26.14%) | 211 (22.62%) |
| Married | 243 (56.51%) | 598 (60.77%) | 2546 (67.53%) | 2944 (73.86%) | 722 (77.38%) |
| **Age Groups** |  |  |  |  |  |
| 18–29 | 124 (28.84%) | 217 (22.05%) | 934 (24.77%) | 832 (20.87%) | 260 (27.87%) |
| 30–41 | 109 (25.35%) | 235 (23.88%) | 971 (25.76%) | 979 (24.56%) | 284 (30.44%) |
| 42–53 | 117 (27.21%) | 240 (24.39%) | 902 (23.93%) | 887 (22.25%) | 194 (20.79%) |
| 54–65 | 47 (10.93%) | 193 (19.61%) | 632 (16.76%) | 709 (17.79%) | 115 (12.33%) |
| + 65 | 33 (7.67%) | 99 (10.06%) | 331 (8.78%) | 579 (14.53%) | 80 (8.57%) |
| **Settlement** |  |  |  |  |  |
| Rural | 33 (7.67%) | 92 (9.35%) | 263 (6.98%) | 394 (9.88%) | 55 (5.89%) |
| Urban | 397 (92.33%) | 892 (90.65%) | 3507 (93.02%) | 3592 (90.12%) | 878 (94.11%) |
| **Job status** |  |  |  |  |  |
| Not employed | 237 (55.12%) | 572 (58.13%) | 2088 (55.38%) | 2374 (59.56%) | 536 (57.48%) |
| Employed | 193 (44.88%) | 412 (41.87%) | 1682 (44.62%) | 1612 (40.44%) | 397 (42.55%) |
| **Education Level** | |  |  |  |  |
| Below high school | 265 (61.63%) | 565 (57.42%) | 2078 (55.12%) | 2351 (58.98%) | 508 (54.45%) |
| High school and above | 165 (38.37%) | 419 (42.58%) | 1692 (44.88%) | 1635 (41.02%) | 425 (45.55%) |
| **Income Groups** | |  |  |  |  |
| Q1 | 154 (35.81%) | 294 (29.88%) | 749 (19.87%) | 743 (18.64%) | 137 (14.68%) |
| Q2 | 126 (29.30%) | 278 (28.25%) | 1032 (27.37%) | 1026 (25.74%) | 248 (26.58%) |
| Q3 | 59 (13.72%) | 153 (15.55%) | 707 (18.75%) | 735 (18.44%) | 162 (17.36%) |
| Q4 | 51 (11.86%) | 149 (15.14%) | 771 (20.45%) | 813 (20.40%) | 199 (21.33%) |
| Q5 | 40 (9.30%) | 110 (11.18%) | 511 (13.55%) | 669 (16.78%) | 187 (20.04%) |
| **Health Satisfaction** | |  |  |  |  |
| Not Satisfied | 242 (56.28%) | 485 (49.29%) | 1520 (40.32%) | 895 (22.45%) | 160 (17.15%) |
| Satisfied | 188 (43.72%) | 499 (50.71%) | 2250 (59.68%) | 3091 (77.55%) | 773 (82.85%) |
| **Income Satisfaction** | |  |  |  |  |
| Not Satisfied | 379 (88.14%) | 845 (85.87%) | 2706 (71.78%) | 1833 (45.99%) | 310 (33.23%) |
| Satisfied | 51 (11.86%) | 139 (14.13%) | 1064 (28.22%) | 2153 (54.01%) | 623 (66.77%) |
| **Social Life Satisfaction** | |  |  |  |  |
| Not Satisfied | 367 (85.35%) | 788 (80.08%) | 2552 (67.69%) | 1748 (43.85%) | 320 (34.30%) |
| Satisfied | 63 (14.65%) | 196 (19.92%) | 1218 (32.31%) | 2238 (56.15%) | 613 (65.70%) |
| **Source of Happiness** | |  |  |  |  |
| Others | 194 (45.12%) | 363 (36.89%) | 1117 (29.63%) | 949 (23.81%) | 317 (33.98%) |
| Health | 236 (54.88%) | 621 (63.11%) | 2653 (70.37%) | 3037 (76.19%) | 616 (66.02%) |
| **Hopeful about the Future** | |  |  |  |  |
| Not hopeful | 330 (76.74%) | 691 (70.22%) | 1309 (34.72%) | 618 (15.50%) | 91 (9.75%) |
| Hopeful | 100 (23.26%) | 293 (29.78%) | 2461 (65.28%) | 3368 (84.50%) | 842 (90.25%) |

**Table A. 4.** Distribution of Life Satisfaction by Happiness Categories in 2021

| **Variable Category** | **Not Happy at all** | **Unhappy** | **Neither Happy nor Unhappy** | **Happy** | **Very Happy** |
| --- | --- | --- | --- | --- | --- |
| **Gender** |  |  |  |  |  |
| Male | 193 (57.61%) | 700 (55.07%) | 1724 (50.38%) | 1918 (42.62%) | 242 (44.40%) |
| Female | 142 (42.39%) | 571 (44.93%) | 1698 (49.62%) | 2582 (57.38%) | 303 (55.60%) |
| **Marital Status** |  |  |  |  |  |
| Not married | 147 (43.88%) | 512 (40.28%) | 1103 (32.23%) | 1071 (23.80%) | 112 (20.55%) |
| Married | 188 (56.12%) | 759 (59.72%) | 2319 (67.77%) | 3429 (76.20%) | 433 (79.45%) |
| **Age Groups** |  |  |  |  |  |
| 18–29 | 109 (32.54%) | 325 (25.57%) | 810 (23.67%) | 853 (18.96%) | 128 (23.49%) |
| 30–41 | 77 (22.99%) | 289 (22.74%) | 840 (24.55%) | 1115 (24.78%) | 161 (29.54%) |
| 42–53 | 72 (21.49%) | 320 (25.18%) | 798 (23.32%) | 1025 (22.78%) | 123 (22.57%) |
| 54–65 | 52 (15.52%) | 216 (16.99%) | 603 (17.62%) | 853 (18.96%) | 74 (13.58%) |
| + 65 | 25 (7.46%) | 121 (9.52%) | 371 (10.84%) | 654 (14.53%) | 59 (10.83%) |
| **Settlement** |  |  |  |  |  |
| Rural | 19 (5.67%) | 112 (8.81%) | 210 (6.14%) | 306 (6.80%) | 21 (3.85%) |
| Urban | 316 (94.33%) | 1159 (91.19%) | 3212 (93.86%) | 4194 (93.20%) | 524 (96.15%) |
| **Job status** |  |  |  |  |  |
| Not employed | 191 (57.01%) | 711 (55.94%) | 1813 (52.98%) | 2620 (58.22%) | 291 (53.39%) |
| Employed | 144 (42.99%) | 560 (44.06%) | 1609 (47.02%) | 1880 (41.78%) | 254 (46.61%) |
| **Education Level** | |  |  |  |  |
| Below high school | 183 (54.63%) | 737 (57.99%) | 1876 (54.82%) | 2646 (58.80%) | 277 (50.83%) |
| High school and above | 152 (45.37%) | 534 (42.01%) | 1546 (45.18%) | 1854 (41.20%) | 268 (49.17%) |
| **Income Groups** | |  |  |  |  |
| Q1 | 85 (25.37%) | 355 (27.93%) | 621 (18.15%) | 773 (17.18%) | 49 (8.99%) |
| Q2 | 95 (28.36%) | 324 (25.49%) | 773 (22.59%) | 959 (21.31%) | 99 (18.17%) |
| Q3 | 68 (20.30%) | 217 (17.07%) | 653 (19.08%) | 891 (19.80%) | 109 (20.00%) |
| Q4 | 41 (12.24%) | 189 (14.87%) | 670 (19.58%) | 865 (19.22%) | 123 (22.57%) |
| Q5 | 46 (13.73%) | 186 (14.63%) | 705 (20.60%) | 1012 (22.49%) | 165 (30.28%) |
| **Health Satisfaction** | |  |  |  |  |
| Not Satisfied | 165 (49.25%) | 600 (47.21%) | 1298 (37.93%) | 997 (22.16%) | 82 (15.05%) |
| Satisfied | 170 (50.75%) | 671 (52.79%) | 2124 (62.07%) | 3503 (77.84%) | 463 (84.95%) |
| **Income Satisfaction** | |  |  |  |  |
| Not Satisfied | 300 (89.55%) | 1113 (87.57%) | 2575 (75.25%) | 2314 (51.42%) | 189 (34.68%) |
| Satisfied | 35 (10.45%) | 158 (12.43%) | 847 (24.75%) | 2186 (48.58%) | 356 (65.32%) |
| **Social Life Satisfaction** | |  |  |  |  |
| Not Satisfied | 283 (84.48%) | 1043 (82.06%) | 2271 (66.36%) | 1967 (43.71%) | 159 (29.17%) |
| Satisfied | 52 (15.52%) | 228 (17.94%) | 1151 (33.64%) | 2533 (56.29%) | 386 (70.83%) |
| **Source of Happiness** | |  |  |  |  |
| Others | 150 (44.78%) | 478 (37.61%) | 1061 (31.01%) | 1146 (25.47%) | 184 (33.76%) |
| Health | 185 (55.22%) | 793 (62.39%) | 2361 (68.99%) | 3354 (74.53%) | 361 (66.24%) |
| **Hopeful about the Future** | |  |  |  |  |
| Not hopeful | 283 (84.48%) | 959 (75.45%) | 1581 (46.20%) | 981 (21.80%) | 79 (14.50%) |
| Hopeful | 52 (15.52%) | 312 (24.55%) | 1841 (53.80%) | 3519 (78.20%) | 466 (85.50%) |

**Table A. 5.** Polychoric Correlation Matrix of Explanatory Variables (2019)

| **Variables** | **Gender** | **Marital Status** | **Settlement** | **Age Groups** | **Job status** | **Education Level** | **Income Groups** | **Health Satisfaction** | **Income Satisfaction** | **Social Life Satisfaction** | **Source of Happiness** | **Hopeful about the Future** |
| --- | --- | --- | --- | --- | --- | --- | --- | --- | --- | --- | --- | --- |
| **Gender** | 1.0000 |  |  |  |  |  |  |  |  |  |  |  |
| **Marital Status** | -0.0458 | 1.0000 |  |  |  |  |  |  |  |  |  |  |
| **Settlement** | -0.0173 | -0.0554 | 1.0000 |  |  |  |  |  |  |  |  |  |
| **Age Groups** | 0.0212 | 0.2792 | -0.1944 | 1.0000 |  |  |  |  |  |  |  |  |
| **Job status** | **-0.5907** | 0.0292 | 0.0637 | -0.3302 | 1.0000 |  |  |  |  |  |  |  |
| **Education Level** | -0.1881 | -0.2797 | 0.3461 | **-0.4653** | 0.3402 | 1.0000 |  |  |  |  |  |  |
| **Income Groups** | -0.0913 | 0.0032 | 0.3346 | -0.1707 | 0.3621 | **0.5208** | 1.0000 |  |  |  |  |  |
| **Health Satisfaction** | -0.2209 | -0.0379 | 0.1218 | -0.3767 | 0.2909 | 0.2744 | 0.1644 | 1.0000 |  |  |  |  |
| **Income Satisfaction** | -0.0255 | -0.0566 | 0.0721 | 0.0321 | 0.0439 | 0.1426 | 0.3098 | 0.3185 | 1.0000 |  |  |  |
| **Social Life Satisfaction** | -0.0456 | -0.0988 | 0.1156 | -0.0189 | -0.0180 | 0.1072 | 0.1491 | 0.3416 | **0.6012** | 1.0000 |  |  |
| **Source of Happiness** | 0.1137 | 0.2058 | -0.0833 | 0.2522 | -0.1107 | -0.2095 | -0.0724 | -0.0870 | 0.0181 | -0.0001 | 1.0000 |  |
| **Hopeful about the Future** | 0.0182 | 0.0452 | -0.0348 | 0.0252 | -0.0327 | 0.0139 | 0.0754 | 0.2889 | 0.4288 | 0.3954 | 0.0621 | 1.0000 |

**Table A. 6.** Polychoric Correlation Matrix of Explanatory Variables (2020)

| **Variables** | **Gender** | **Marital Status** | **Settlement** | **Age Groups** | **Job status** | **Education Level** | **Income Groups** | **Health Satisfaction** | **Income Satisfaction** | **Social Life Satisfaction** | **Source of Happiness** | **Hopeful about the Future** |
| --- | --- | --- | --- | --- | --- | --- | --- | --- | --- | --- | --- | --- |
| **Gender** | 1.0000 |  |  |  |  |  |  |  |  |  |  |  |
| **Marital Status** | -0.0316 | 1.0000 |  |  |  |  |  |  |  |  |  |  |
| **Settlement** | -0.0040 | -0.0144 | 1.0000 |  |  |  |  |  |  |  |  |  |
| **Age Groups** | -0.0020 | 0.4078 | -0.1579 | 1.0000 |  |  |  |  |  |  |  |  |
| **Job status** | **-0.5738** | 0.0362 | 0.1126 | -0.2725 | 1.0000 |  |  |  |  |  |  |  |
| **Education Level** | -0.1309 | -0.3394 | 0.3306 | **-0.4704** | 0.2906 | 1.0000 |  |  |  |  |  |  |
| **Income Groups** | -0.0424 | 0.0291 | 0.3689 | -0.1343 | 0.3290 | **0.4877** | 1.0000 |  |  |  |  |  |
| **Health Satisfaction** | -0.1292 | -0.0921 | 0.0026 | -0.3030 | 0.2346 | 0.2480 | 0.1723 | 1.0000 |  |  |  |  |
| **Income Satisfaction** | 0.0242 | -0.0559 | 0.0015 | 0.0595 | 0.0329 | 0.1063 | 0.3606 | 0.3484 | 1.0000 |  |  |  |
| **Social Life Satisfaction** | -0.0049 | -0.0788 | 0.0026 | 0.0153 | -0.0163 | 0.0548 | 0.1578 | 0.3585 | **0.6126** | 1.0000 |  |  |
| **Source of Happiness** | 0.1494 | 0.2596 | -0.1036 | 0.2808 | -0.1118 | -0.2030 | -0.0609 | -0.0595 | 0.0418 | 0.0593 | 1.0000 |  |
| **Hopeful about the Future** | 0.0350 | 0.1072 | -0.0354 | 0.0632 | 0.0024 | -0.0367 | 0.1447 | 0.2997 | 0.4742 | 0.4047 | 0.1274 | 1.0000 |

**Table A. 7.** Polychoric Correlation Matrix of Explanatory Variables (2021)

| **Variables** | **Gender** | **Marital Status** | **Settlement** | **Age Groups** | **Job status** | **Education Level** | **Income Groups** | **Health Satisfaction** | **Income Satisfaction** | **Social Life Satisfaction** | **Source of Happiness** | **Hopeful about the Future** |
| --- | --- | --- | --- | --- | --- | --- | --- | --- | --- | --- | --- | --- |
| **Gender** | 1.0000 |  |  |  |  |  |  |  |  |  |  |  |
| **Marital Status** | -0.0498 | 1.0000 |  |  |  |  |  |  |  |  |  |  |
| **Settlement** | -0.0287 | 0.0038 | 1.0000 |  |  |  |  |  |  |  |  |  |
| **Age Groups** | 0.0062 | 0.3499 | -0.1662 | 1.0000 |  |  |  |  |  |  |  |  |
| **Job status** | **-0.5955** | 0.0426 | 0.1495 | -0.3258 | 1.0000 |  |  |  |  |  |  |  |
| **Education Level** | -0.1554 | -0.3421 | 0.4047 | **-0.4922** | 0.3488 | 1.0000 |  |  |  |  |  |  |
| **Income Groups** | -0.0756 | 0.0299 | 0.4155 | -0.1713 | 0.3757 | **0.5065** | 1.0000 |  |  |  |  |  |
| **Health Satisfaction** | -0.2089 | -0.0731 | 0.1345 | -0.3311 | 0.2776 | 0.2877 | 0.2068 | 1.0000 |  |  |  |  |
| **Income Satisfaction** | -0.0236 | -0.0315 | 0.0558 | 0.0493 | 0.0812 | 0.1137 | 0.3188 | 0.3503 | 1.0000 |  |  |  |
| **Social Life Satisfaction** | 0.0006 | -0.0755 | 0.1290 | 0.0011 | -0.0201 | 0.0851 | 0.1526 | 0.3548 | **0.6309** | 1.0000 |  |  |
| **Source of Happiness** | 0.1387 | 0.2845 | -0.0094 | 0.3156 | -0.1252 | -0.2461 | -0.0815 | -0.0879 | 0.0058 | 0.0180 | 1.0000 |  |
| **Hopeful about the Future** | 0.0310 | 0.1334 | -0.0172 | 0.1323 | -0.0511 | -0.0571 | 0.1033 | 0.2363 | 0.4458 | 0.3935 | 0.1395 | 1.0000 |

**Table A. 8.** Variance Inflation Factors (VIF) for Explanatory Variables

| **Variable** | **2019** | | **2020** | | **2021** | |
| --- | --- | --- | --- | --- | --- | --- |
|  | **VIF** | **1/VIF** | **VIF** | **1/VIF** | **VIF** | **1/VIF** |
| **Gender** (Female) | 1.250 | 0.800 | 1.230 | 0.813 | 1.260 | 0.794 |
| **Marital Status** (Married) | 1.270 | 0.787 | 1.360 | 0.735 | 1.350 | 0.741 |
| **Age Groups** *(Reference: 18–29)* | | | | | | |
| 30–41 | 1.900 | 0.526 | 1.940 | 0.515 | 1.960 | 0.510 |
| 42–53 | 1.990 | 0.503 | 2.020 | 0.495 | 2.070 | 0.483 |
| 54–65 | 1.900 | 0.526 | 1.880 | 0.532 | 1.950 | 0.513 |
| +65 | 1.760 | 0.568 | 1.650 | 0.606 | 1.770 | 0.565 |
| **Settlement** (Urban) | 1.040 | 0.962 | 1.060 | 0.943 | 1.070 | 0.935 |
| **Job status** (Employed) | 1.490 | 0.671 | 1.440 | 0.694 | 1.520 | 0.658 |
| **Education Level** (≥ High school) | 1.390 | 0.719 | 1.390 | 0.719 | 1.420 | 0.704 |
| **Income** *(Lowest 20% income group)* | | | | | | |
| Q2 | 1.630 | 0.613 | 1.790 | 0.559 | 1.840 | 0.543 |
| Q3 | 1.730 | 0.578 | 1.670 | 0.599 | 1.820 | 0.549 |
| Q4 | 1.680 | 0.595 | 1.850 | 0.541 | 1.950 | 0.513 |
| Q5 | 2.000 | 0.500 | 1.940 | 0.515 | 2.250 | 0.444 |
| **Health** (Satisfied) | 1.220 | 0.820 | 1.180 | 0.847 | 1.190 | 0.840 |
| **Income** (Satisfied) | 1.330 | 0.752 | 1.390 | 0.719 | 1.350 | 0.741 |
| **Social Life** (Satisfied) | 1.260 | 0.794 | 1.270 | 0.787 | 1.290 | 0.775 |
| **Source of Happiness** (Health) | 1.050 | 0.952 | 1.070 | 0.935 | 1.090 | 0.917 |
| **Hopeful about the Future** (Hopeful) | 1.120 | 0.893 | 1.140 | 0.877 | 1.140 | 0.877 |
| **Mean** | **1.501** | **0.698** | **1.515** | **0.691** | **1.572** | **0.672** |

1. The answers are updated every year due to inflation. Therefore. the 2019 answers are used as an example.
